# Comparative Efficacy of Quadratus Lumborum Muscle Energy Technique with Gluteus Medius Strengthening Versus Gluteus Medius Strengthening Alone in Sacroiliac Joint Dysfunction: A Randomized Controlled Trial

**DOI:** 10.3390/diagnostics14212413

**Published:** 2024-10-30

**Authors:** Rabail Rani Soomro, Hossein Karimi, Syed Amir Gilani

**Affiliations:** 1 Department of Physiotherapy, Sindh Institute of Physical Medicine and Rehabilitation, Karachi 74200, Pakistan; 2 University Institute of Physical Therapy, University of Lahore, Lahore 54000, Pakistan; dr.hossein.karimi@gmail.com (H.K.); profgilani@gmail.com (S.A.G.); 3 Faculty of Health Sciences, Istanbul Gelisim University, Istanbul 34310, Turkey; 4 Department of Rehabilitation Sciences, Green International University Lahore, Lahore 55150, Pakistan

**Keywords:** gluteus medius muscle, muscle energy technique, quadratus lumborum, sacroiliac joint dysfunction

## Abstract

Background: Pain in the sacroiliac joint is the most prevalent and often overlooked. The sacroiliac joints are thought to be sources of pain in roughly 10% to 25% of patients with chronic lower back pain. Due to the biomechanical nature of the joint, muscle imbalance is the most important cause of sacroiliac joint dysfunction. The hamstring and gluteus medius are the primary muscles involved in postural dysfunction-related muscle imbalance; however, the quadratus lumborum’s role in the compensatory mechanism is becoming more apparent, and its potential for treatment in conjunction with gluteus medius strengthening has not yet been investigated. Gluteus medius exercises, along with conventional treatment, are routinely given to patients with sacroiliac joint dysfunction; however, the aim of this study is to explore the additional effects of the muscle energy technique (MET) on the quadratus lumborum along with strengthening of the gluteus medius on pain, disability and quality of life of patients with sacroiliac joint dysfunction. Methods: Using a computer-generated random number table, seventy patients with unilateral sacroiliac joint pain were divided equally and randomly into two groups. Prior to initiating treatment, baseline measurements were taken using a hand-held dynamometer, visual analog scale (VAS), Oswestry Disability Index (ODI-U) and short form 36-item survey (SF-36v2) to assess strength, pain, functional disability and quality of life, respectively. Over the course of four weeks, all patients received twelve sessions, and both the pre- and post-intervention outcome measures were documented. Results: After 4 weeks of treatment, both groups showed statistically significant (*p* < 0.005) mean improvements in muscle strength, pain, disability and quality of life before and after intervention. However, the mean improvements in post-intervention on a dynamometer, VAS, ODI and SF-36 were better in the MET with exercise group (METGME) as compared to the conventional group with exercise (CTGME), with a larger effect size. Conclusions: The muscle energy technique, applied to the quadratus lumborum in combination with gluteus medius strengthening, is more effective clinically and significantly in improving pain, disability and quality of life in comparison to conventional treatment of sacroiliac joints with gluteus medius exercises.

## 1. Introduction

SIJ pain resulting from abnormal motion of the joint is known as SIJ dysfunction (SIJD). Thirty percent of patients with LBP endorse pain originating from the sacroiliac joint (SIJ) [1]. It can also be referred to as a biomechanical issue that can involve muscle imbalance [2]. Correct muscle activation allows for normal load transmission across the lumbopelvic region; hence, the lack of movement at the sacroiliac joint due to improper muscle activations and forces is considered a dysfunction [3]. Various factors can cause muscle imbalances around the sacroiliac joint, including poor posture, repetitive movements, muscle weakness, tightness and trauma [4]. One of the major muscles that have gained attention in recent times and impact many in the management of sacroiliac joint dysfunction is the gluteus medius [5]. A weak gluteus medius impairs its primary role as the pelvic stabilizer, which in turn creates instability in the lateral hip and excessive adduction during walking. The hip will rise to lessen foot striking on the lateral foot and reduce the degree of adduction [6]. As a result, the quadratus lumborum works compensatory with excessive hip hiking, which leads to its hypertonicity and can become a foundation of pain caused by overactivity, and subsequent trigger points refer to radiating pain into the sacroiliac joint region [7]. Additionally, a recent study says that the myofascial pain of the QL muscle is among the most common musculoskeletal pathologies in patients with lower back pain, and this pain in the deep fibers of the muscle extends to the sacroiliac joint and the lower part of the hip [8].

The current literature suggests that specific gluteus medius strengthening exercises can help improve pelvic stability and reduce strain on the sacroiliac joint, resulting in an improvement in sacroiliac joint dysfunction [5,8]. Quadratus lumborum is a highly prevalent muscle involved in lower back pain that is associated with the sacroiliac joint, and many studies have shown evidence of different treatment techniques on the quadratus lumborum that result in improvement [9,10,11]. However, the muscle energy technique on the quadratus lumborum is considered to be the most useful pain relief technique for lower back pain [12,13]. Despite the high prevalence of quadratus lumborum tightness seen in patients having lower back pain, not many studies validate the effect of its length in subjects having sacroiliac joint-associated lower back pain. The solo effects of gluteus medius exercises [14] and quadratus lumborum [15] release have been explored, but the combined effects of quadratus lumborum release and gluteus medius strengthening in subjects having sacroiliac joint dysfunction have not yet been investigated. Therefore, the aim of this study is to compare the effectiveness of the quadratus lumborum muscle energy technique with gluteus medius strengthening exercises versus gluteus medius strengthening alone in the management of sacroiliac joint dysfunction.

## 2. Methods

### 2.1. Study Setting and Participants

This randomized controlled trial was conducted at the Sindh Institute of Physical Medicine and Rehabilitation Karachi from December 2019 to August 2022. Ethical approval was obtained from the Institutional Review Board (IRB) of the University of Lahore, and the trial was registered at www.ClinicalTrial.gov with registration identification NCT04161443. A total of 70 patients aged 30 to 50 with unilateral nonspecific sacroiliac joint pain, having at least 3 positive SIJ pain provocative tests out of 5, were recruited for the study. The inclusion criteria also comprised both genders, a pain history greater than six weeks, a sacroiliac joint (SIJ) diagnostic scoring cut-off point of 4, and a VAS cut-off point of mild to moderate. The exclusion criteria were radiating pain with sensory or motor deficits, history of fracture or any spinal surgery, trauma, any dysfunction of the hip or knee, any systemic disease and pregnancy.

### 2.2. Sample Size

It was estimated using an OpenEpi sample size calculator for the mean difference, version 3.01. After inserting the mean and SD of the MET (1.06 ± 0.25) and control groups (2.06 ± 1.33) at a 95% confidence interval, we required at least *n* = 30 participants for this study, 15 in each group. However, we added 20% to the sample size to compensate for potential attrition, therefore recruiting 42 participants in our study, 21 in each treatment group. The sample size was calculated using online software (http://www.openepi.com/SampleSize/SSMean.htm, accessed on 1 October 2018), and the formula used for this calculator is *n* = (Zα/2 + Zβ)^2^ × 2 × σ^2^/d^2^. Moreover, to avoid statistical error and assuming the burden of lower back pain, a sample size of 70 patients was taken, which was equally divided into 35 for each group [16].

### 2.3. Initial Assessment

The initial assessment was conducted in two phases. Phase I included a diagnosis of patients with sacroiliac joint dysfunction. All the patients coming to the outpatient department with the primary complaint of nonspecific back pain associated with the sacroiliac joint were diagnosed with sacroiliac joint dysfunction by the rehab physician based on their history, physical examination and assessment. The patients were later sent to the physiotherapy department at Sindh Institute of Physical Medicine and Rehabilitation for further inclusion in the study and treatment. Phase II of the assessment was carried out by trained physiotherapists with more than 5 years of experience in musculoskeletal physiotherapy. In phase II of the initial assessment, the BMI was taken, and the demographic data were gathered through the screening proforma. Comprehensive subjective and objective assessments were carried out with special consideration given to the inclusion and exclusion criteria. Those patients who met the inclusion criteria were recruited for the study after their approval via informed consent.

### 2.4. Randomization and Allocation

**Figure 1:**
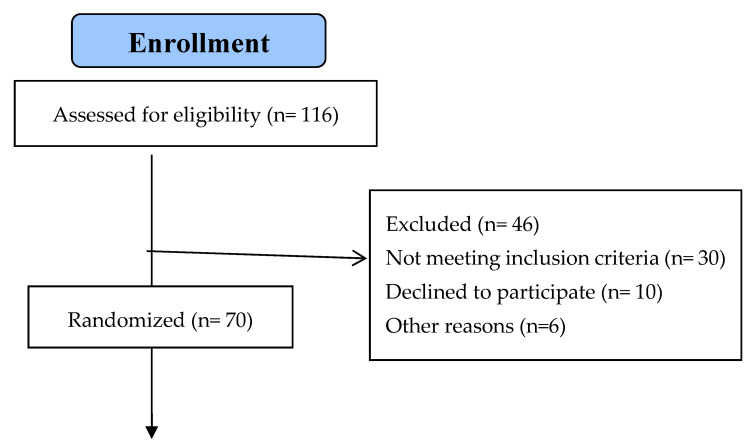

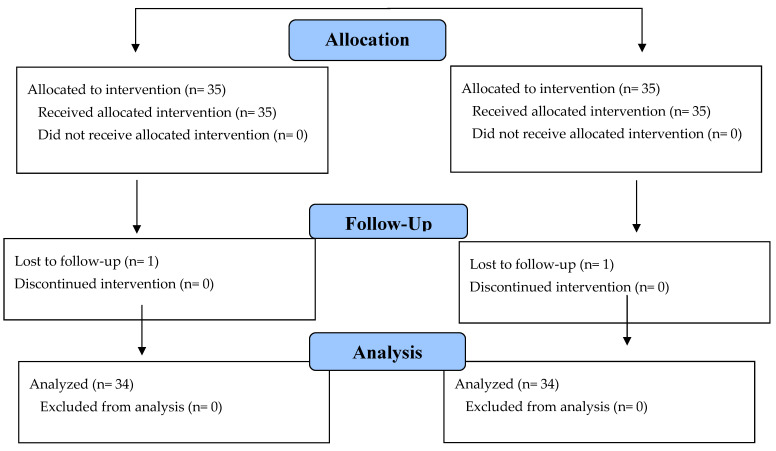
Flow chart of the recruitment, randomization and follow-up of patients.

Using a random sampling method, a computer-generated random number table was used to carry out the randomization. Those numbers were sealed into envelopes, and the primary investigator unlocked the envelopes containing those numbers in order to designate the patients in the allocated group. After the initial assessment of 116 patients, 70 patients were randomly and equally allocated to two groups. A total of 35 patients were in the MET with gluteus medius exercise group (METGME), and 35 patients were in conventional treatment with the gluteus medius exercise group (CTGME). The CONSORT flow diagram shows how the participants were assigned to these groups (Figure 1).

### 2.5. Consent Form and Questionnaire

The consent form clearly stated the study objectives and any possible harms and benefits. Informed consent, as well as the screening proforma and all outcome measures, were available in both English and Urdu versions for ease of understanding. Patients were taken into confidence about their confidentiality and were informed that their participation in this study was completely voluntary and that they could withdraw at any time from the study. The treatment was provided by an experienced physiotherapist and was totally free of cost.

### 2.6. Masking

This study was a double-blinded randomized controlled trial in which the patients were unaware of the treatment given, and the outcomes assessor was blinded to the allocation of the patients to either of the groups.

### 2.7. Intervention

The patients in the experimental and control groups received conventional physical therapy treatment for sacroiliac joint dysfunction, i.e., ultrasound, hamstring stretches and gluteus medius exercises; however, only the experimental group was given the muscle energy technique for the quadratus lumborum in addition to conventional treatment. Both groups received the treatment for a total of 4 weeks. Each patient completed a total of twelve treatment sessions (i.e., three sessions per week on alternate days at the physical therapy outpatient department at Sindh Institute of Physical Medicine and Rehabilitation). A total of 70 patients (35 in each group) were assigned randomly to the following groups, amongst which 2 of the patients dropped out, 1 in each group.

#### 2.7.1. Control Group (CTGME)

Conventional physical therapy treatment was comprised of ultrasound, exercises and stretching, including posture awareness and a home exercise plan. The ultrasound was applied over the painful area of the sacroiliac joint with circular movements of the probe head for 5 min using the intensity of 1.3 W/cm^2^ and frequency of 1 MHZ in a continuous mode. Exercises of the gluteus medius included isolated exercises for each fiber. With the knee extended while side-lying, the anterior gluteus medius exercise involved hip adduction. The exercise for the middle gluteus medius was a wall-press exercise. The exercise for the posterior gluteus medius was pelvic drop exercise, which is intended to preserve an appropriate pelvic tilt [14]. Active hamstring stretches were given as 3 repetitions for 30 s. For all exercises, three sets of 30 repetitions were performed during the session, and the same was given at home for 4 weeks.

#### 2.7.2. Experimental Group (METGME)

Along with the treatment provided to the control group, patients in the experimental group also received the muscle energy technique. Using post-isometric relaxation of MET, the experimental group underwent additional isometric contraction six times per session for a total of twelve sessions. The contraction was held for five seconds and followed by a five-second rest period. The therapist stood waist-level behind the side-lying patient to perform the procedure. The patient held the upper end of the table firmly with their upper arm extended over their head. As they inhaled, they abducted their upper leg until the therapist felt strong quadratus lumborum activity, which is normally felt at an elevation of about thirty degrees. In this way, the patient held their leg (and their breath) isometrically, allowing gravity to act as resistance. After 5 s of contraction, the patient’s leg hung a little behind him. To target the activation of different fibers, this action was repeated alternately with a raised leg in front and behind the trunk. The therapist firmly held the pelvis with both hands, with fingers interlocking over the crest of the pelvis and leaned back to take out all slack and to ease the pelvis away from the lower ribs during exhalation. After making sure the patient had fully relaxed for five seconds, this was conducted by passively advancing to a new restricted barrier without stretching in the direction of the long axis of the abducted leg. On exhaling, the patient moved to a new barrier.


**Interventional Groups**

**Experimental Group**
**(Treatment Time 30 min)**

**Control Group**
**(Treatment Time 25 min)**

MET quadratus lumborumUltrasoundGluteus medius exercisesHamstring stretch

UltrasoundGluteus medius exercisesHamstring stretch
Posture advice and home exercise programDuration of treatment, 4 weeksThree visits per week Total of 12 sessionsAssessment at baseline and final assessment at the twelfth session.

### 2.8. Outcome Measures

#### 2.8.1. VAS

VAS is a validated tool used for assessing pain. It is a subjective measure used for both acute and chronic pain. It is a 10 cm straight line. One side of the straight line has minimum/no pain, and the other side has the worst pain from left to right. The patient marked his/her pain on the line. The score was assessed by the therapist, where 0–3.4 cm is mild, 3.5–7.4 cm is moderate and 7.5–10 cm on a 10 cm line is considered severe pain [17].

#### 2.8.2. ODI

The Oswestry Disability Index is the “gold standard” self-administered questionnaire for determining functional impairment in lower back pain. Ten areas covering social and sexual life, other personal activities and the level of pain are included. There are six alternative responses in each area, which are to be graded on a 0–5 scale. A higher degree of functional impairment is indicated by the higher scores. The final score, which is typically expressed as a percentage, is 50. For the ODI, there is currently no accepted minimal clinically relevant difference (MCID) score. The ODI’s MCID varies, with changes of 17, 10, 6 and 5 points [18]. With good to moderate validity and great reliability in patients with lumbar radiculopathy, the Oswestry Disability Index (ODI-U) in Urdu was utilized [19]. According to reports, patients with lumbar radiculopathy had an MDC of roughly 6 points on a 0–50 scale.

#### 2.8.3. SF-36

The short-form 36-item questionnaire (SF-36), Urdu version, was used to assess patients’ quality of life. A 36-item patient self-assessment of general health yields scores on two component summary measures and eight health domain scales. The SF-36v2 measures eight different aspects of health: mental health (MH), role-functioning (RF), physical function (PF), bodily pain (BP), general health (GH), vitality (VT), social function (SF), role-emotional (RE) and general health (GH). By combining these eight dimensions, two summary scores can be obtained: the PCS (physical component summary score) and the MCS (mental component summary score) [20]. These summaries are only available with a licensed version of SF36v2. The license to use the questionnaire, SF-36v2 QOL Urdu version, was officially taken from Optum Insight Life Sciences, Inc. (Boston MA, USA), with license number QM053455. In this study, the scores were calculated through an algorithm using software provided by Optum Insight Life Sciences. The RAND 36-Item Health Survey, which is disseminated by RAND, is used in common practice as it does not require licensing and is free to use [21]. This comprises items identical to the SF-36; however, the suggested scoring system differs from the SF-36’s scoring. The SF36v2 used in this study is, therefore, a highly valid, responsive, reliable and internally consistent tool for assessing the health status and quality of life of patients, particularly those with lower back pain [20].

#### 2.8.4. Dynamometer

Due to the fact that hip abduction is the principal function of the gluteus medius (GMe) muscle, its strength was evaluated using the Microfet2 hand-held dynamometer (MicroFET 2, Hoogan Health Industries, West Jordan, UT, USA). Increased strength was indicated by a greater HHD value, whereas muscular weakening was represented by a lower value [22]. This hand-held dynamometer has shown excellent inter-rater and intra-rater reliability in measuring the isometric strength of the gluteus medius [23].

### 2.9. Frequency and Duration of Treatment

A total of 12 sessions were carried out over a period of 4 weeks with a frequency of 3 sessions each week, and the duration of each session was half an hour. Both groups received treatment with postural advice and a home exercise program.

### 2.10. Harm and Adverse Events

No harm or any adverse event was reported during the period of the trial.

### 2.11. Data Analysis Procedure

Data were stored and analyzed using IBM-SPSS version 23.0. Counts with percentages were reported on gender, duration of symptoms and diagnostic test (joint distraction, compression, Gaenslen maneuver, FABER and thigh thrust) between the two studied groups. The mean and standard deviation were reported for the gluteus medius muscle strength testing by the dynamometer reading KGF unit, visual analog scale, Oswestry Disability Index scoring, physical functioning scale, role physical scale, bodily pain scale, general health, vitality scale, social functioning, role-emotional, mental health, physical component summary, mental component summary, mental health enhanced score and SF-6D health utility index score at baseline and the twelfth session of study. A between-group comparison was made using an independent sample *t*-test, whereas inter-group analysis was made using a paired sample *t*-test. For qualitative parameters, the Pearson Chi-square test was used to study the association, and *p*-values less than 0.05 were considered statistically significant.

## 3. Results

Seventy patients were found eligible according to the selection criteria. In each group, one patient dropped out and was not available for the post-treatment assessment. The withdrawal details are mentioned in Figure 1. In Table 1, the baseline characteristics of all participants are summarized.

In the control group, most of the participants (64.7%) had a duration of symptoms between 6 to 8 weeks, some (26.5%) had between 8 to 10 weeks, and very few (9.8%) had symptoms between 10–12 weeks. However, in the experimental group, there were almost an equal number of participants (32.4%) between 6 to 8 weeks, (32.4%) between 8 to 10 weeks and (35.3%) between 10–12 weeks. The dominant side of sacroiliac joint dysfunction among participants in the experimental and control groups was almost equal, with 50% in the experimental group being right dominant and 50% being left dominant in the control group.

Moreover, in the experimental group, 55.9% of the participants had three positive sacroiliac joint provocative tests, 35.3% had four and 8.8% had all five tested positive. In the control group, 35.3% had three positive sacroiliac joint provocative tests, 52.9% had four and 11.8% had all five test positive. There was a significant difference between the two groups in terms of duration of symptoms (*p*-value 0.01), but there was no significant difference (*p*-value > 0.05) between the two groups in terms of gender, age, BMI, sacroiliac joint diagnostic score, sacroiliac joint dysfunction dominant side and sacroiliac joint provocative test. (Table 1).

Both groups showed statistically significant mean improvements in muscle strength, pain, disability, and quality of life before and after intervention. However, mean improvements in post-intervention on the dynamometer, VAS, ODI and SF36v2 were better in the MET with the exercise group as compared to conventional treatment with the exercise group.

In the MET with exercise group, the baseline means for the dynamometer, SF36 (PCS and SF-6D health utility index score) values improved from 6.99, 28.34 and 0.44 to 12.71, 56.41 and 0.67, respectively, in the 4 weeks’ time period. Not much improvement was detected in the MCS, with the means in the experimental group ranging from 40.06 to 39.53 and in the control group from 39.68 to 43.13, respectively. Moreover, in the experimental group, a significant reduction was detected in pain on the visual analog scale from means of 43.32 to 4.73, disability on the Oswestry Disability Index scoring from 48.11 to 12.03 and quality of life on the mental health enhanced score from 16.89 to 11.74. (Table 2).

After 4 weeks of intervention, there were significant differences in the inter- and intra-group analyses. There were statistically significant mean differences among both the MET with exercise group and exercise alone conventional group on the dynamometer, VAS, ODI and SF36v2 quality of life at baseline and 4 weeks of intervention within and between groups. There was no statistical mean difference, with an insignificant *p*-value (*p*-value 0.89) of the role-emotional component of SF36 between the experimental and control groups after 12 sessions. Moreover, the results were statistically insignificant, with a *p*-value of 0.23 for social functioning within the control group before and after 12 sessions of intervention. Table 3.

Furthermore, there is a larger MCID for the experimental group compared to the control group, which shows more effectiveness of treatment in the experimental group from baseline to the twelfth session as compared to the control group.

Table 4 reports the minimally clinically important difference (MCID) for the control and experimental group samples using each scale of treatment, and the results show the MCID for the experimental/control groups for gluteus medius muscle strength testing by dynamometer reading KGF unit as (4.3/1.3), visual analog scale as (2.2/2.3), Oswestry Disability Index scoring as (2.5/3), physical functioning scale as (4.6/0.6), role physical scale as (5.8/0.8), bodily pain scale as (2.8/0.7), general health as (2.7/1.3), vitality scale as (1.7/0.8), social functioning as (1.9/1.1), role-emotional as (1.1/0.8), mental health as (1.3/0.8), physical component summary as (6.3/1.2), the mental component summary as (1.4/1.1), and for the mental health enhanced score (1.3/0.8).

## 4. Discussion

This study investigated the additional effects of the muscle energy technique upon the quadratus lumborum along with gluteus medius strengthening exercises comparatively to conventional treatment with exercises in the management of sacroiliac joint dysfunction. The comparative effects of this study suggest that more improvements occurred in the MET with the gluteus medius exercise group in improving pain, functional disability and quality of life. Additionally, MET with exercise is more statistically significant with a greater mean difference and is clinically significant with a larger MCID in contrast to the conventional treatment with exercise group following 4 weeks of intervention. Not only this, but the experimental group also had a greater effect on muscle strengthening via the dynamometer compared to the control group. The magnitude of the effects in this present study is large and consistent with the recent systematic reviews [8,24]. These systematic reviews emphasize an understanding of exercise for the gluteus medius and state that compensatory activation of the quadratus lumborum for a weaker Gmed can lead to lateral instability and compromised motions such as lumbar spine lateral flexion or lateral tilt of the pelvis [24]. Consequently, while recommending Gmed strengthening exercises, the associated activation of synergistic muscles or compensatory movement should be taken into account. This idea is supported by the fact that the current study used MET primarily for quadratus lumborum muscle release to relieve soft tissue tension, which was then followed by gluteus medius strengthening exercises. A similar study was conducted on individuals with lower back pain who had trigger points in their QL muscles. The results showed that MET significantly reduced pain and functional impairment when compared to the strain-counter-strain approach [25]. In a different study, patients with sacroiliac joint dysfunction were treated with MET, and the results showed that MET considerably lowers the anterior pelvic tilting angle and pain level in people with chronic SIJ but has no effect on sacroiliac stiffness [15]. Nevertheless, in this current study, the VAS, ODI and SF36 were utilized to measure the study’s outcomes; the pelvic tilt angle and joint stiffness were not. In a number of studies, MET is compared with different techniques such as chiropractic manipulation [26], dry needling [27], myofascial release and stretching [12], interferential therapy [11], transcutaneous electrical muscle stimulation [28] and manipulation [29], in which MET has been superior to all other treatments. Moreover, the evidence shows that MET is also used in combination with exercises versus exercises alone. In a recent study, MET was compared with aerobic exercise, and it was concluded that the effects of the muscle energy technique were found to be more pronounced than those of aerobic exercise [30]. Furthermore, another study supported the preference for MET with exercise compared to MET alone. This study suggested that, when combined with supervised exercise, MET has an additional positive impact on function and disability reduction in individuals with chronic nonspecific lower back pain, as compared to subjects who were simply subjected to MET [31]. The exercises given in this study were general exercises, such as abdominals, superman, cat camel bridging, etc., whereas contrary to the current study, exercises are very specific and isolated so as to focus on gluteus medius strengthening. The findings of the present study suggested improvement not only in pain and disability but also in the strengthening of the gluteus medius muscle observed on HHD, being clinically significant with a larger effect size. Similar to the current investigation, a previous study comparing GMED sub-division exercises to sacroiliac joint mobilization demonstrated a significant decrease in pelvic tilt, with an equal number of positive provocation test findings in both groups [32]. The anterior, middle and posterior fibers of the GMED subdivisions are the main pelvic stabilizers and are necessary to preserve the pelvis’ and lower limb’s regular movement patterns [14,32]. The primary outcome tool for GMED strengthening in the current study was HHD, a relatable measure of muscle strength, rather than the provocative test or pelvic tilt angle. Provocative tests were only used during the screening phase of the study, where most patients had three or four positive test results. In another study, daily activities and the lumbar stiffness disability index were used as outcome tools, along with VAS and ODI, to observe the effects of MET on SI joint dysfunction [33]. Supporting the present study results, MET had assisted in relieving the level of pain and disability in daily activities but could not alleviate lumbar stiffness. Moreover, the study duration was short-term, as the outcome tools were evaluated 24 h and one week later after treatment. Contrary to that, our study duration was 4 weeks, having a total of 12 sessions of treatment. The duration of the effects of MET is also debatable in the literature, as some studies favor short-term effects and some long-term effects. A pilot randomized clinical study was conducted on a small sample size and concluded that both the MET and MDT techniques have short-term effects on sacroiliac joint dysfunction after only four treatment sessions [34]. Following this study, another study found the therapeutic effects of MET before, after and after 24 h, supporting the evidence of the short-term benefits of MET. It was further found that the analgesic effect of MET can continue for up to 24 h in other points of the body [35]. Nonetheless, certain studies also indicate that MET has a remarkable impact over prolonged periods of intervention [36,37,38]. Tahir et al. compared the effects of MET with stabilizing exercise using the same treatment length as the current study—four weeks or twelve sessions in total—three times a week [5]. Their significant findings were similar to those of the current study. In the current study, the SF36v outcome tool was used as a measure to observe the improvement in the quality of life of both the experimental and control groups. The results suggest a major improvement in the physical component summary and an average improvement in the mental component summary but no improvement in the role-emotional component of the SF36 between the experimental and control groups after 12 sessions. Improved functional status and reduced pain are thought to contribute to a better quality of life and a sense of well-being. However, the inconsequential outcome of role-emotional could be due to the fact that emotions are not continuous and are linked to events. Fareeha et al. also mentioned in their study the reason behind this could be due to the fact that post-treatment measurements were taken immediately after the four-week intervention— the same as the current study’s duration and recommended long-term follow-up to improve all domains of QOL [19].

MET is an active muscle release technique and has been used for a long time to treat osteoarticular illness by numerous researchers, including Chaitow and Crenshaw [39], Greenman [40] and Frank [41].

The Golgi tendon organ (GTO) makes the antagonist muscle contract more easily while inhibiting the contraction of the agonist muscle, enabling the muscle to be stretched farther and more easily [35]. The series of studies by these researchers validate the use of MET to increase the extensibility of soft tissues to correct pelvic and lumbar dysfunctions. This method, however, in the current study, was used to correct the alignment of sacroiliac joint dysfunction by releasing the tension in the major quadratus lumborum muscle. According to another study, a combination of tightness and weakness in the muscles causes an imbalance that changes the alignment of a certain body segment. These changes in alignment lead to the additional contraction of tissues that are already shortened and place stress on the joint surfaces from weight bearing. Through compensatory feedback loops, this imbalance weakens segmental control [42]. In the present study, we assumed a tightness of the quadratus lumborum and a weakness of the gluteus medius, and hence, the MET on the QL and strengthening of the Gmed brought significant change clinically and statistically on all outcome measure tools. It is further advisable to use the MET technique first before using other rehabilitation techniques (like strengthening), as Rowlands asserted that agonist and antagonist muscles are stimulated concurrently by MET, which appears to lessen pain perception [43]. These findings lend further credence to the idea of gluteus medius strengthening exercises performed after applying MET.

### Limitations

This study is limited to the results on the basis of the VAS for pain intensity, the dynamometer for muscle strength, the ODI questionnaire for assessing functional disability and the SF36 for quality of life only. This study is limited to patients with unilateral nonspecific sacroiliac joint pain with a pain history of more than 6 weeks. The initial assessment was limited to manual pain provocative tests.

## 5. Conclusions

The muscle energy technique on the quadratus lumborum, alongside strengthening the gluteus medius, is clinically and significantly more effective in improving pain, disability and quality of life in comparison to conventional treatment of sacroiliac joint dysfunction with gluteus medius exercise. Future studies are needed; however, the current study recommends that the quadratus lumborum should not be overlooked while treating sacroiliac joint dysfunction.

## Figures and Tables

**Table 1:** Baseline characteristics of studied samples.

Characteristics		Study Groups				*p*-Value
		Experimental Group (*n* = 34)		Control Group (*n* = 34)		
		N	%	N	%	
Gender	Male	17	50	17	50	0.99
	Female	17	50	17	50	
Age (years) ^¥^	Mean (±SD)	42.08	5.85	42.65	5.86	0.69
BMI (kg/m^2^) ^¥^	Mean (±SD)	25.92	3.43	26.66	3.44	0.38
Sacroiliac joint diagnostic score 0–9 points at baseline ^¥^	Mean (±SD)	5.73	1.19	5.68	1.22	0.85
Duration of symptoms	6 to 8 weeks	11	32.4	22	64.7	0.01 *
	8 to 10 weeks	11	32.4	9	26.5	
	10 to 12 weeks	12	35.3	3	9.8	
Sacroiliac joint dysfunction dominant side	Right side	17	50	17	50	0.99
	Left side	17	50	17	50	
Sacroiliac joint provocative tests at baseline	3 test positive	19	55.9	12	35.3	0.23
	4 test positive	12	35.3	18	52.9	
	All 5 test positive	3	8.8	4	11.8	
	Positive	10	27.0	4	12.9	

* *p* < 0.05 was considered statistically significant using the Pearson Chi-square test; ¥: *p*-value was calculated using an independent sample *t*-test.

**Table 2:** Description of studied parameters at baseline and fourth week in experimental and control group samples.

Parameters	Baseline	12th Session	Baseline	12th Session
	Exp	Exp	Control	Control
Gluteus medius muscle strength testing by dynamometer reading KGF unit	6.99(±1.36)	12.71(±1.33)	7.39(±1.36)	9.75(±2.12)
Visual analog scale 0–100 mm line	43.32(±17.78)	4.73(±6.86)	57.35(±12.31)	27.29(±14.2)
Oswestry Disability Index scoring	48.11(±15.96)	12.03(±4.48)	63.23(±13.76)	37.74(±11.17)
Physical component summary	28.34(±4.26)	56.41(±4.62)	34.44(±3.78)	37.8(±4.2)
Mental component summary	40.06(±4.57)	39.53(±5.5)	39.68(±4.65)	43.13(±4.92)
Mental health enhanced score	16.89(±4.08)	11.74(±4.19)	16.26(±3.97)	14.08(±4.04)
SF-6D health utility index score	0.44(±0.05)	0.67(±0.05)	0.5(±0.07)	0.54(±0.06)

**Table 3:** Inter- and intra-group analyses of studied parameters.

Parameters	At Baseline Experimental vs. Control ^¥^		At 12th Session Experimental vs. Control ^¥^		Experimental Baseline vs. 12th Session ^£^		Control Baseline vs. 12th Session ^£^	
	MD	*p*-Value	MD	*p*-Value	MD	*p*-Value	MD	*p*-Value
Gluteus medius muscle strength testing by dynamometer reading KGF unit	−0.4	0.24	2.96	<0.01 *	−5.72	<0.01 *	−2.36	<0.01 *
Visual analog scale	−14.03	<0.01 *	−22.56	<0.01 *	38.59	<0.01 *	30.06	<0.01 *
Oswestry Disability Index scoring	−15.12	<0.01 *	−25.71	<0.01 *	36.08	<0.01 *	25.49	<0.01 *
Physical functioning scale	−14.95	<0.01 *	59.91	<0.01 *	−84.86	<0.01 *	−10	<0.01 *
Role physical scale	−15.8	<0.01 *	39.27	<0.01 *	−67.57	<0.01 *	−12.5	<0.01 *
Bodily pain scale	−15.72	<0.01 *	24.13	<0.01 *	−47.97	<0.01 *	−8.12	<0.01 *
General health	0.24	0.90	13.44	<0.01 *	−19.46	<0.01 *	−6.26	<0.01 *
Vitality scale	−8.47	<0.01 *	8.81	<0.01 *	−22.12	<0.01 *	−4.84	<0.01 *
Social functioning	−4.79	0.19	13.6	<0.01 *	−21.62	<0.01 *	−3.23	0.23
Role-emotional	−2.88	0.44	0.57	0.89	−15.55	<0.01 *	−12.1	<0.01 *
Mental health	−1.61	0.53	6.75	0.02 *	−14.33	<0.01 *	−5.97	<0.01 *
Physical component summary	−6.1	<0.01 *	18.61	<0.01 *	−28.07	<0.01 *	−3.36	<0.01 *
Mental component summary	0.38	0.73	−3.6	0.02 *	0.53	0.53	−3.45	<0.01 *
Mental health enhanced score	0.63	0.52	−2.34	<0.01 *	5.15	<0.01 *	2.18	<0.01 *
SF-6D health utility index score	−0.06	<0.01 *	0.13	<0.01 *	−0.23	<0.01 *	−0.04	<0.01 *

MD: mean difference; * mean difference was considered statistically significant with *p* < 0.05; ¥: *p*-value based on independent sample *t*-test; £: *p*-value based on paired sample *t*-test.

**Table 4:** Minimally clinically important difference (MCID).

Scales	MCID	
	Experimental	Control
Gluteus medius muscle strength testing by dynamometer reading KGF unit	4.3	1.3
Visual analog scale	2.2	2.3
Oswestry Disability Index scoring	2.5	3.0
Physical functioning scale	4.6	0.6
Role physical scale	5.8	0.8
Bodily pain scale	2.8	0.7
General health	2.7	1.3
Vitality scale	1.7	0.8
Social functioning	1.9	1.1
Role-emotional	1.1	0.8
Mental health	1.3	0.8
Physical component summary	6.3	1.2
Mental component summary	1.4	1.1
Mental health enhanced score	1.3	0.8
SF-6D health utility index score	3.6	0.9

## Data Availability

The data are available from the corresponding author upon reasonable request.
