# Correction: Soomro et al. Comparative Efficacy of Quadratus Lumborum Muscle Energy Technique with Gluteus Medius Strengthening Versus Gluteus Medius Strengthening Alone in Sacroiliac Joint Dysfunction: A Randomized Controlled Trial. *Diagnostics* 2024, *14*, 2413

**DOI:** 10.3390/diagnostics15212710

**Published:** 2025-10-27

**Authors:** Rabail Rani Soomro, Hossein Karimi, Syed Amir Gilani

**Affiliations:** 1 Department of Physiotherapy, Sindh Institute of Physical Medicine and Rehabilitation, Karachi 74200, Pakistan; 2 University Institute of Physical Therapy, University of Lahore, Lahore 54000, Pakistan; dr.hossein.karimi@gmail.com (H.K.); profgilani@gmail.com (S.A.G.); 3 Faculty of Health Sciences, Istanbul Gelisim University, Istanbul 34310, Turkey; 4 Department of Rehabilitation Sciences, Green International University Lahore, Lahore 55150, Pakistan

## Additional Affiliation

All authors wish to add a new affiliation [1]. The updated affiliation should include the following: University Institute of Physical Therapy, University of Lahore, Lahore 54000, Pakistan.

## Error in Institutional Review Board Statement

In the published publication, there was an error in Institutional Review Board Statement. The corrected one is: This study followed the Declaration of Helsinki. Ethical approval was obtained from the Institutional Review Board, University of Lahore, with ref: IRB-UOL-FAHS/373-VII/2018 on 20 September 2018. The study’s protocol was prospectively registered on ClinicalTrials.gov Identifier: NCT04161443.

 

The authors state that the scientific conclusions are unaffected. This correction was approved by the Academic Editor. The original publication has also been updated.
